# Recent progress in practical applications of a potential carotenoid astaxanthin in aquaculture industry: a review

**DOI:** 10.1007/s10695-022-01167-0

**Published:** 2023-01-06

**Authors:** Samia Elbahnaswy, Gehad E. Elshopakey

**Affiliations:** 1 Department of Internal Medicine, Infectious and Fish Diseases, Faculty of Veterinary Medicine, Mansoura University, Mansoura, 35516 Egypt; 2 Department of Clinical Pathology, Faculty of Veterinary Medicine, Mansoura University, Mansoura, 35516 Egypt

**Keywords:** Antioxidant, Astaxanthin, Disease resistance, Immune system, Pigmentation, Stress

## Abstract

Astaxanthin is the main natural C40 carotenoid used worldwide in the aquaculture industry. It normally occurs in red yeast *Phaffia rhodozyma* and green alga *Haematococcus pluvialis* and a variety of aquatic sea creatures, such as trout, salmon, and shrimp. Numerous biological functions reported its antioxidant and anti-inflammatory activities since astaxanthin possesses the highest oxygen radical absorbance capacity (ORAC) and is considered to be over 500 more times effective than vitamin E and other carotenoids such as lutein and lycopene. Thus, synthetic and natural sources of astaxanthin have a commanding influence on industry trends, causing a wave in the world nutraceutical market of the encapsulated product. In vitro and in vivo studies have associated astaxanthin’s unique molecular features with various health benefits, including immunomodulatory, photoprotective, and antioxidant properties, providing its chemotherapeutic potential for improving stress tolerance, disease resistance, growth performance, survival, and improved egg quality in farmed fish and crustaceans without exhibiting any cytotoxic effects. Moreover, the most evident effect is the pigmentation merit, where astaxanthin is supplemented in formulated diets to ameliorate the variegation of aquatic species and eventually product quality. Hence, carotenoid astaxanthin could be used as a curative supplement for farmed fish, since it is regarded as an ecologically friendly functional feed additive in the aquaculture industry. In this review, the currently available scientific literature regarding the most significant benefits of astaxanthin is discussed, with a particular focus on potential mechanisms of action responsible for its biological activities.

## Highlights

- Beneficial use of astaxanthin as a feed supplement in cultured aquatic species.
- Screening of astaxanthin in pigmentation, growth and immunity enhancement, inflammatory response, and disease resistance of aquatic species.
- Astaxanthin prevents several diseases associated with oxidative stress in aquatic animals.

## Introduction

Carotenoids are the most common class of lipid-soluble pigments that feature a broad group of molecules that are naturally produced by plants and many photosynthetic organisms. These molecules are defined as natural antioxidants, protecting the cells from oxidative stress mediated by either light, free radical-mediated peroxidation, or singlet oxygen (Merhan 2017). Carotenoids have been widely applied in the pharmaceutical, cosmetic, and feed industries owing to their positive biological characteristics in humans and animals (Maoka 2011, 2012, 2015; Milani et al. 2017; Rodriguez-Concepcion et al. 2018). Given these properties, several healthy effects have been promoted by carotenoids, such as those on the immune response, reproduction, lipid metabolisms, photoprotection in the skin, and chronic disease prevention such as diabetes, cardiovascular disease, hypertension, atherosclerosis, cancer, and inflammation (Maoka 2015; Komatsu et al. 2017; Rodriguez-Concepcion et al. 2018; Bae et al. 2020; Britton 2020; Donoso et al. 2021). Among them, astaxanthin (AX), an orange-reddish ketocarotenoid pigment with superior antioxidative activity, has been shown to have the highest oxygen radical absorbance capacity in comparison with other carotenoids (Nakagawa et al. 2011; Merhan 2017). Besides, functional supplements from natural sources could be considered safe agents for infectious diseases and environmental stressor prevention in farmed fish and crustaceans and subsequently humans (Fletcher 1997; Gatlin et al. 2007; Nakano et al. 2018). Thus, astaxanthin confers a significant impact on improvements in global fish farming owing to the accumulation of residual antibiotics in fish tissues and side effects on fish health (Elia et al. 2014; Nakano et al. 2018). Considerable work has strengthened the demand for the production and utilization of natural sources of astaxanthin as a pigment coloring agent which currently covers most of the world markets (Higuera-Ciapara et al. 2006; Seabra and Pedrosa 2010; Rahman et al. 2016). Also, the key role in the pigmentation of aquatic animals is played by astaxanthin (Rahman et al. 2016). Given its unique features, the use of astaxanthin has attracted considerable interest in the last years in aquatic animal rearing and is expected to be a feasible pathway to the sustainable development of aquaculture (Nakano and Wiegertjes 2020; Lu et al. 2021).

The AX can naturally synthesize from a wide variety of sources, such as bacteria, red yeast *Phaffia rhodozyma*, microalgae, *Haematococcus pluvialis*, *Chlorella vulgaris*, *Chlorella zofingiensis*, and *Chlorococcum* sp. which have biodegradable, no drug resistance, and an environment friendly in a variety of fish species. Also, AX can be obtained indirectly in our diet by consuming crustaceans (e.g., copepods, shrimp, and krill) and Salmonidae (e.g., salmon, rainbow trout) species, whose diets include natural sources of astaxanthin (Liu et al. 2016; Wang et al. 2018b; Lu et al. 2021).

Commercial synthesis of astaxanthin is currently the most cost-effective that dominated by synthetically derived astaxanthin in over 95% of the feed market (> 95%) (Lim et al. 2018; Stachowiak and Szulc 2021). According to Grand View Research, the global astaxanthin market value was estimated at USD 1.0 billion in 2019 and expecting to witness a compound annual growth rate of 16.2% from 2019 to 2027 to reach USD 3398.8 million by 2027, owing to only using natural astaxanthin in the pharmaceutical, cosmetic, and food industries and its multifunctional health benefits and safety (Silva et al. 2021; Stachowiak and Szulc 2021).

Astaxanthin takes part a crucial role in antioxidant, anti-inflammatory, immunity enhancement, and growth promotion (Jagruthi et al. 2014; Li et al. 2014; Lim et al. 2018). Previous studies have shown that dietary AX could reduce oxidative stress and also could improve the immune response, disease resistance, and growth performance of different fish and crustaceans (Jagruthi et al. 2014; Han et al. 2018; Lim et al. 2018; Li et al. 2020). Thus, the use of astaxanthin in aquaculture can boost the immunity of aquatic animals, decreasing mortality and preventing antibiotic abuse (Alishahi et al. 2015; Lu et al. 2021).

This article is to review the recent progress of available scientific works of literature regarding the most significant activities of astaxanthin as an economically valuable product in aquaculture, including its antioxidative, immunity response, and anti-inflammatory properties, its protective effects on reproduction, skin pigmentation, infectious diseases, and stress tolerance.

## Astaxanthin as a valuable biologically active compound

### Chemical structure and biochemistry of astaxanthin

**Fig. 1:**
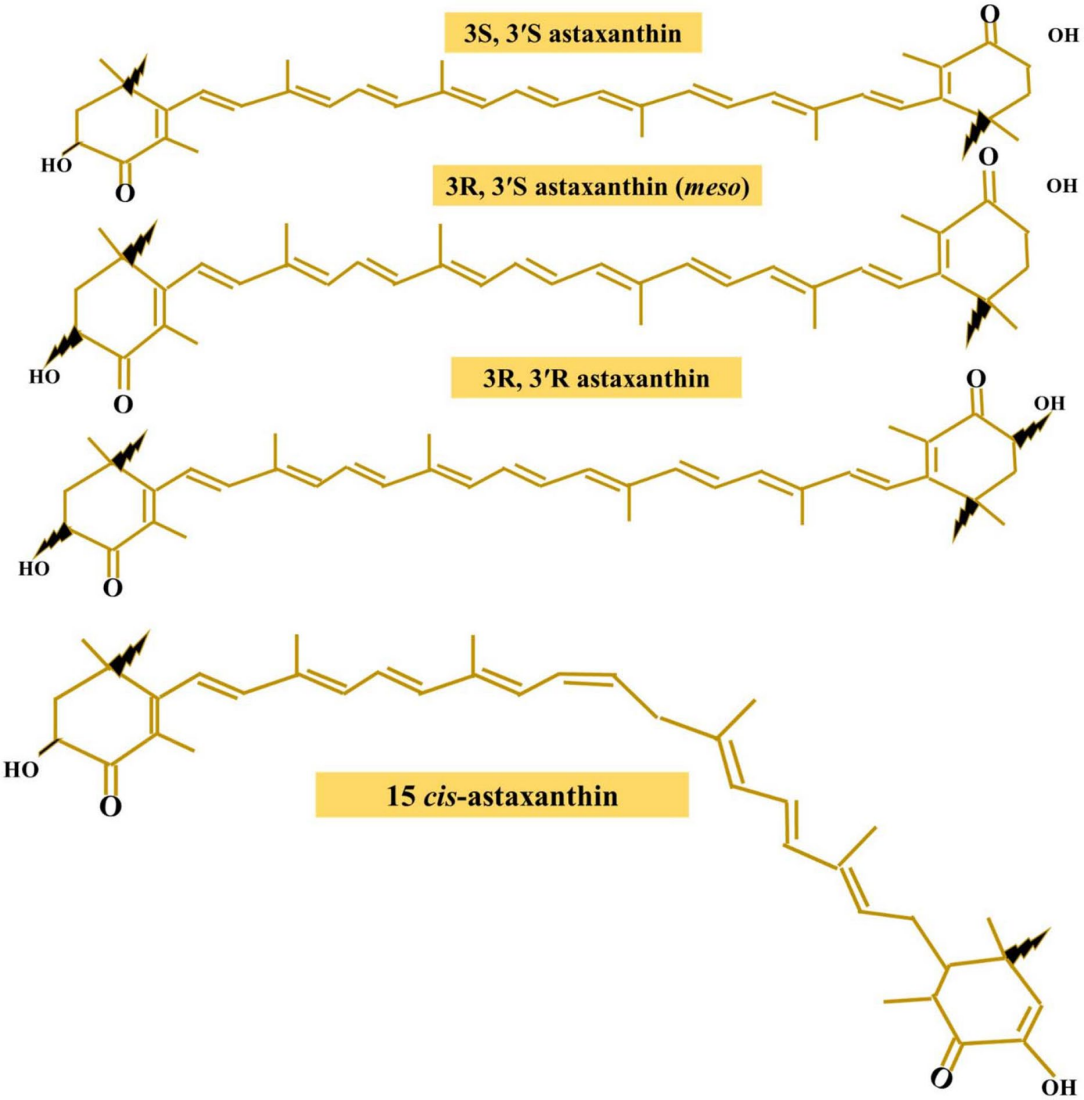
Chemical planner structure of astaxanthin configurational isomers and a geometric *Cis* isomer

The major structure of carotenoids is composed of hydrocarbons of 40 carbon atoms, including two terminal ring systems linked by a chain of conjugated double bonds or polyene systems. In general, carotenoids can be singled out as two groups based on their structural chemical elements: carotenes, which are only composed of carbon and hydrogen, and xanthophylls, which contain oxygen derivatives. In the xanthophylls, oxygen is exhibited as hydroxyl (OH) groups, keto-moieties (C = O) groups, or as a combination of both, as displayed in astaxanthin (AX) (Fig. 1) (Britton 1995; Higuera-Ciapara et al. 2006; Seabra and Pedrosa 2010; Yuan et al. 2011; Lin et al. 2016). Structural features of (OH) and (C = O) groups in each ionone ring elucidate some of the astaxanthin properties, such as esterification ability, a more polar nature, and a major antioxidant capacity (Liu and Osawa 2007; Guerra et al. 2012). The presence of a polyene system provides the carotenoids with their distinctive molecular structure and chemical and light-absorbing characteristics. Every double polyene bond can be existed in two configurations, as *cis* or *trans* geometric isomers. Thermodynamically, *trans*-isomers are more stable than other *cis*-isomers. As known, most carotenoids, in specific astaxanthin, are found as *trans* isomers in nature; however, they could be isomerized to another form when exposed to light, heat, acid, or metal ions (Liu and Osawa 2007). Besides geometric isomers which contain two stereogenic carbon atoms at the *C*-3 and *C*-3′ in each molecule, astaxanthin exists in three stereoisomers: two enantiomers (3*R*, 3′*R*- and 3*S*, 3′*S*-astaxanthin) and an inactive meso form (3*R*, 3′*S*-astaxanthin) (Turujman et al. 1997; Ambati et al. 2014). Naturally, 3*S*, 3′*S*-astaxanthin is the most abundant stereoisomer as a variety of organisms produce astaxanthin in different stereoisomeric ratios (Grewe et al. 2007; Wang et al. 2008). In particular, crustaceans contain three types of optical isomers (Higuera-Ciapara et al. 2006). In the natural environment, astaxanthin is mainly esterified with fatty acids (monoesters and diesters) or conjugated with proteins, such as in crustacean exoskeleton and salmon muscle, giving molecular stability, whereas astaxanthin could also be found without esterification as a free-ester astaxanthin form (Turujman et al. 1997; Østerlie et al. 1999; Storebakken et al. 2004). On the other hand, synthetic astaxanthin includes a racemic mixture of stereoisomers and is not found as a free-ester astaxanthin form (Yuan et al. 1997; Higuera-Ciapara et al. 2006). Another substantial source of AX is the marine bacterium *Agrobacterium aurantiacum* (*A*. *aurantiacum*), which synthesized (3*S*,3′*S*)-astaxanthin and (3*S*,3′*R*)-adonixanthin (4-ketozeaxanthin) from *β*-carotene through two hydroxylation steps at *C*-3 and 3′ and oxidation steps at *C*-4 and 4′ (Yokoyama et al. 1994, 1995; Yokoyama and Miki 1995; Wang et al. 2017).

**Fig. 2:**
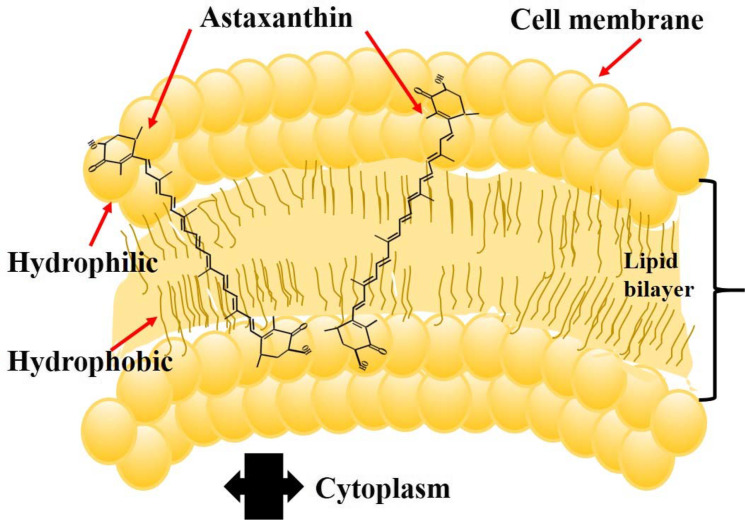
Schematic view of the astaxanthin molecule position at the cell membrane

Astaxanthin has centrally located conjugated double bonds, hydroxyl, and keto-groups (Higuera-Ciapara et al. 2006; Ambati et al. 2014). These double bonds give its red coloration and robust antioxidant activity through donating the electrons and reacting with free radicals to convert them to more stabilized products in a wide variety of living organisms (Guerin et al. 2003; Seabra and Pedrosa 2010; Donoso et al. 2021). Indeed, AX has both lipophilic and hydrophilic properties, as it can usually link with the integral part (inside and outside) of the complex membrane structure (Fig. 2) and can be incorporated into liposome phospholipid bilayers; thus, AX exhibited the highest antioxidant efficiency when compared to other carotenoids, such as *β*-carotene, lycopene, and vitamin C (Miki 1991; Britton 1995, 2008; McNulty et al. 2008; Yamashita 2015).

### Astaxanthin sources

Natural microorganisms, particularly algae, fungi, yeast, and bacteria, are constituted the primary natural sources of astaxanthin (Table 1). Besides, the astaxanthin-containing organisms that are consumed by aquatic animals for the acquisition of glamorous coloration pile astaxanthin in the tissues of aquatic organisms. For example, aquatic zooplanktons fed on marine algae (rich in beta-carotene, fucoxanthin, and diatoxanthin) are in turn converting *β*-carotene, which accumulated from marine algae to astaxanthin in their body; consequently, they are ingested by nautical fish (e.g., salmonids and trouts) and crustacean organisms (e.g., shrimp, crabs, crayfish, lobsters, and krill) at higher nutritive levels (Johnson and An 1991; Lim et al. 2018). Synthetic astaxanthin is commercially obtained by either chemical composition (Li et al. 2011; Cheng et al. 2016) or natural microbial resources, such as red yeast *Xanthophyllomyces dendrorhous* (formerly *Phaffia rhodozyma*) (Rodríguez-Sáiz et al. 2010; Hara et al. 2014; Dursun and Dalgıç, 2016) and green microalga *Haematococcus pluvialis* (*H*. *pluvialis*) (Cheng et al. 2016; Wang et al. 2016; Li et al. 2019). The leading role is undoubtedly achieved by *H*. *pluvialis* which is one of the most promising sources of natural astaxanthin, and so many studies have investigated the best conditions to synthesize and extract astaxanthin from *H*. *pluvialis* (Ambati et al. 2014; Shah et al. 2016; Zhao et al. 2019). Furthermore, other significant sources of astaxanthin include the flesh of wild and farmed marine fish, such as salmonids and trout. However, considerable variations were assayed in the muscle astaxanthin content among species. For instance, astaxanthin concentrations in wild *Oncorhynchus* species varied from 3 mg kg^−1^ flesh in chum salmon *Oncorhynchus keta* up to 38 mg kg^−1^ flesh in sockeye salmon *Oncorhynchus nerka* (EFSA 2005). As well, astaxanthin contents in wild and farmed Atlantic salmon *Salmo salar* were reported as 3–10 mg kg^−1^ flesh and 1–9 mg kg ^−1^ flesh, respectively (EFSA 2005). Besides, astaxanthin concentrations were significantly higher in fresh salmon compared to pouch packaged and canned products (Sutliff et al. 2020). Therefore, salmonid fillets can be served as a good dietary source of natural astaxanthin.

**Table 1:** Different natural sources of astaxanthin

Sources	Astaxanthin (%) on a dry weight basis	References
Microalgal source		
Chlorophyceae		
* Botryococcus braunii*	0.01	Grung and Metzger (1994)
* Chlorococcum sp.*	0.2	Zhang et al. (1997)
* Chloromonas nivalis*	0.004	Remias et al. (2005)
* Eremosphera viridis*	4	Leya et al. (2009)
* Chlorella vulgaris*	0.55	Safi et al. (2014)
* Chlorella zofingiensis*	0.68	Orosa et al. (2001)
* Chlorella zofingiensis*	0.001	Wang et al. (2008)
* Haematococcus pluvialis*	4	Lee and Ding (1994)
* Haematococcus pluvialis (K-0084)*	3.8	Aflalo et al. (2007)
* Haematococcus pluvialis (AQSE002)*	3.4	Olaizola (2000)
* Haematococcus pluvialis (K-0084)*	2.7	Wang et al. (2013)
* Haematococcus pluvialis*	3.8	Ranga Rao et al. (2010)
* Neochloris wimmeri*	0.6	Orosa et al. (2000)
* Protosiphon botryoides*	1.4	Orosa et al. (2000)
* Scotiellopsis oocystiformis*	1.1	Khandual (2019)
* Scenedesmus obliquus*	0.3	Qin et al. (2008)
* Tetraselmis* sp.	0.23	Raman and Mohamad (2012)
* Nannochloropsis salina* * Nannochloropsis oculata*	1.5 2.5	Zanella and Vianello (2020)
Algal source		
Florideophyceae		
* Catenella repens*	0.02	Banerjee et al. (2009)
Ulvophyceae		
* Enteromorpha intestinalis*	0.02	Banerjee et al. (2009)
* Ulva lactuca*	0.01	Banerjee et al. (2009)
Bacterial source		
Alphaproteobacteria		
* Agrobacterium aurantiacum*	0.01	Yokoyama et al. (1995)
* Paracoccus carotinifaciens*	2.2	Bories et al. (2007)
Fungal source		
Labyrinthulomycetes		
* Thraustochytrium* sp.	0.2	Yamaoka (2008)
Tremellomycetes		
* Xanthophyllomyces dendrorhous (JH)*	0.5	Kim et al. (2005)
* Xanthophyllomyces dendrorhous (VKPM Y2476)*	0.5	de la Fuente et al. (2010)
Crustacean		
Malacostraca		
* Pandalus borealis*	0.12	EFSA (2005)
* Pandalus clarkia*	0.015	Meyers and Bligh (1981)

**Fig. 3:**
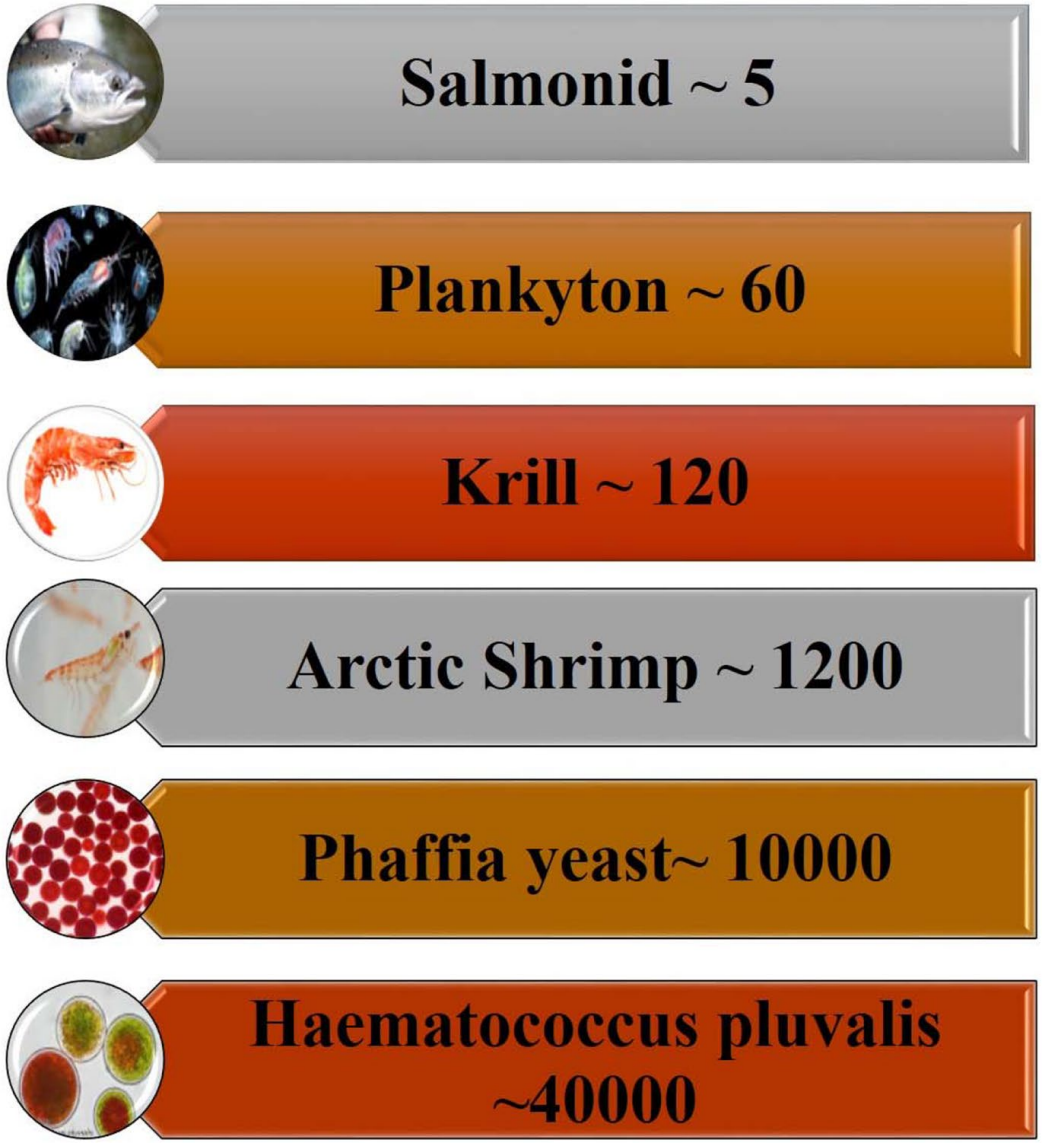
Astaxanthin concentrations from natural sources are approximately presented from Wikipedia web site

Among crustaceans, shrimps have been widely studied due to their capacity in producing astaxanthin and so they are important dietary sources of astaxanthin (Niamnuy et al. 2008; Ju et al. 2009; Tume et al. 2009). The astaxanthin contents of 1.41 mg 100 g^−1^ and 1.69 mg 100 g^−1^ were found in the muscles of wild *Penaeus semisulcatus* and *Metapenaues monoceros* shrimps, respectively (Yanar et al. 2004). Cultured *Litopenaeus vannamei* (*L*. *vannamei*) fed a basal diet, which contained 2.24 mg of astaxanthin 100 g^−1^ (Ju et al. 2009). Also, dried *Penaeus indicus* (*P*. *indicus*) shrimp had 6.16 mg of astaxanthin 100 g^−1^ (Niamnuy et al. 2008). The Antarctic krill *Euphausia superba* (*E*. *superba*) is an excellent source of astaxanthin diester (55–64%), astaxanthin monoester (25–35%), and astaxanthin (7–8%), especially in the carapace, flesh, and eyes of *E*. *superba*. The amounts of AX in these organs are contained 1.13 mg 100 g^−1^, 1.06 mg 100 g^−1^, and 90.82 mg 100 g^−1^, respectively (Yamaguchi et al. 1983; Maoka et al. 1985). As well, Pacific krill possesses a higher concentration of AX (Koomyart et al. 2017). Additionally, astaxanthin concentrations are mostly detected in the processed wastes of the cephalothorax, abdominal epidermal layer, and abdominal exoskeleton (shells), particularly those ranging from 4.79 mg 100 g^−1^ in *P*. *indicus* to 9.17 mg 100 g^−1^ in *Xiphopenaeus kroyeri* (De Holanda and Netto 2006; Sachindra et al. 2007; Seabra and Pedrosa 2010). On the other hand, red porgy skin (*Pagrus pagrus*) contains higher astaxanthin levels in fish fed *H*. *pluvialis* (4.89 mg 100 g^−1^) than in the fish’s skin fed synthetic astaxanthin (2.91 mg 100 g^−1^). Based on these data, *H*. *pluvialis* provides adequate concentrations of esterified astaxanthin to imbue the skin of red porgy more efficiently which may be indicated by the higher intestinal solubility and easier incorporation of astaxanthin esters into mixed micelles when compared with synthetic, unesterified astaxanthin (Tejera et al. 2007). In general, the primary natural sources of astaxanthin in high concentrations are displayed in Fig. 3 (Ekpe et al. 2018).

### Natural astaxanthin vs synthetic astaxanthin

Many differences are documented between synthetic astaxanthin and natural astaxanthin. Firstly, synthetic astaxanthin is cheaper than natural microalgal astaxanthin since microalgal cultivation and harvesting are cost-consuming. Secondly, synthetic astaxanthin is mostly unesterified while microalgal astaxanthin is esterified (Ambati et al. 2014; Su et al. 2020). Thirdly, synthetic astaxanthin and microalgal astaxanthin contain different geometrical and optical isomers (Su et al. 2020). Previous research assayed that microalgal astaxanthin could be better than synthetic astaxanthin in astaxanthin accumulation, safety, and potential nutritive quality of Chinese mitten crab (*Eriocheir sinensis*) (Yang et al. 2017; Su et al. 2020). Besides, synthetic astaxanthin is markedly inferior to algal natural astaxanthin as an antioxidant (Capelli et al. 2013). Thus, natural astaxanthin from algae and aquatic animals has shown better benefits than synthetic astaxanthin.

### Synthetic mechanisms of astaxanthin

**Fig. 4:**
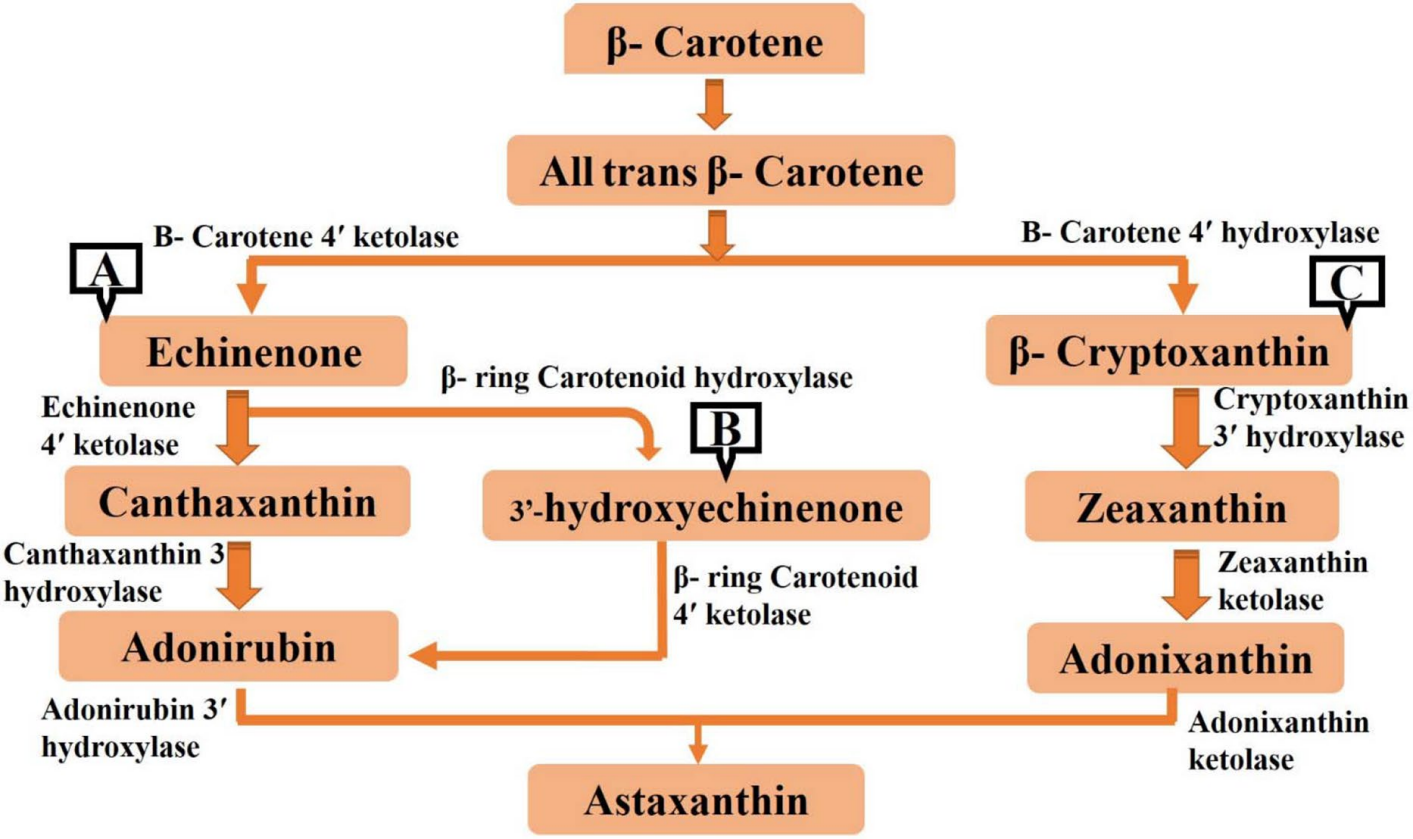
Synthesis pathways of microalgal astaxanthin from *Haematococcus pluvialis* (A), *Scenedesmus obliquus* (B), and *Chlorella zofingiensis* (C)

Astaxanthin production has relied mostly on most microalgal strains, such as *Chlorella zofingiensis*, *Haematococcus pluvialis*, and *Scenedesmus obliquus*. Generally, no obvious morphological changes are observed during the cultivation of green-colored microalgae (Ranjbar et al. 2008). Under adverse conditions, the microalgae cells are transformed into resting cysts, in which microalgae growth is prohibited but the survival efficiency of algal cells is intensified (Kobayashi 2003). In the resting stage, the blood-red color of microalgal cells and astaxanthin content originated in a harsh environment (Ranjbar et al. 2008). Therefore, astaxanthin synthesis in microalgal strains can be regarded as a self-protection mechanism, which enhances the survival of algal cells at the expense of microalgal biomass accumulation. *β*-carotene forms a general precursor for astaxanthin from microalgal cells. The precursor is catalyzed by the enzymatic activity of *β*-carotene ketolase and hydroxylase, resulting in metabolic intermediates canthaxanthin and zeaxanthin, respectively (Rajesh et al. 2017). Therefore, the synthesis process of astaxanthin is performed through different pathways according to microalgal species and enzymatic activities of *β*-carotene (Fig. 4) (Li et al. 2008; Qin et al. 2008). Besides, the natural contents of three geometric isomers (all-*trans*, 9-*cis*, and 13-*cis*-astaxanthin) of astaxanthin differ in microalgae. For instance, the content of all-*trans* astaxanthin is higher than that of cis astaxanthin in *H*. *pluvialis* and *Chlorella zofingiensis*; however, *cis*-astaxanthin has much higher antioxidative properties (Liu and Osawa 2007). In recent years, astaxanthin productivity can be improved by genetic modification technology (Lu et al. 2021).

Under the natural environment, the astaxanthin synthesis in microalgae is too low to encounter the market demand. Therefore, many studies reported production technologies to promote astaxanthin productivity and alleviate the conflict between produced microalgal biomass and astaxanthin synthesis. Many external conditions could be modified to impact the trophic stages of microalgae and so promote the formation of the resting cyst for improving astaxanthin cultivation. As previously reported, astaxanthin synthesis in algal cells is favored by the supply of glucose, salt, nitrogen deficiency, high-intensity illumination, and the addition of trace elements (Fábregas et al. 1998; Janchot et al. 2019; Han et al. 2020; Lu et al. 2021). Under industrial conditions, one-stage and two-stage processes are regarded in the production of astaxanthin. In the one-stage process, some essential nutrients, such as carbon, phosphorus, and nitrogen, are provided into culture media to promote microalgae growth. After that, a harsh environment, such as nutrient depletion and pH change, could dramatically modify the water environment for microalgae cells to transform into resting cysts and the astaxanthin synthesis pathway is activated. Moreover, some inducers (e.g., nitrogen and glucose) are implemented together with microalgae inoculation to enhance its growth. The lower production cost of astaxanthin is one of the disadvantages of the one-stage process. Besides, some inducers would negatively impact algae growth, such as observed in a previous study that nitrogen would hinder the protein synthesis in algal cells and further decrease microalgae growth (Del Río et al. 2008; Mao et al. 2018; Han et al. 2020). As well, environmental changes may adversely impact glucose concentration, which is favorable to astaxanthin synthesis in algal cells (Li et al. 2008).

Two-stage cultivation is employed to overcome the disadvantages of the one-stage process as it mainly consists of a growth stage and an induction stage. The growth of microalgae cells is provided via sufficient nutrients, whereas the induction stage requires specific conditions to promote the transformation of algal cells to resting cysts (Fábregas et al. 2001; Zhu et al. 2021). Therefore, a two-stage process is a more effective strategy to improve astaxanthin production in microalgae (Affenzeller et al. 2009; Lu et al. 2021). As previously reported, the astaxanthin yield reached 4.0% of dry weight in the two-stage cultivation model, while the astaxanthin yield was only 0.8% of dry weight in the one-stage cultivation model (Aflalo et al. 2007).

Genetic modification (GM) has become one of the recent technologies for improving astaxanthin productivity as the genes regulating astaxanthin synthesis have been characterized (Huang et al. 2013; Lu et al. 2021). Previous reports have transferred the genes related to astaxanthin synthesis into tobacco and tomato by using GM technology, obtaining novel plants and fruits enriched with astaxanthin (Hasunuma et al. 2008; Huang et al. 2013). Genetic improvement of *H*. *pluvialis* strains was previously performed to produce astaxanthin using classical mutagenesis, resulting in production of various mutants of *H*. *pluvialis* that have higher astaxanthin accumulation capacity (Hu et al. 2008; Hong et al. 2012; Gómez et al. 2013). Besides, genetic engineering using transformations of *H*. *pluvialis* chloroplast and its nuclear genomes was recently achieved by vector transformation of transgenes into the nuclear genome (5′ or 3′ end) of the endogenous dominant selection marker, phytoene desaturase (pds) variant (Gutierrez et al. 2012; Sharon-Gojman et al. 2015; Shah et al. 2016). However, up to now, the application of these technologies in astaxanthin synthesis is mainly conducted in lab research. Therefore, the astaxanthin synthetized by genetic engineering technology has not been widely used in aquaculture.

### Challenges and opportunities in astaxanthin scale-up

To our knowledge, the high market price of astaxanthin is about 2000 USD per kilogram, which is mainly attributed to the high production cost of microalgae biomass. Thus, its price still may not be affordable to many aquaculture factories, however microalgal astaxanthin is much cheaper than astaxanthin from ocean fisheries (Onorato and Rösch 2020; Sui et al. 2020). Besides, the low content of astaxanthin in biomass is another item, causing the high production cost. Previous documents elucidated that astaxanthin contents in dry biomass of *H*. *pluvialis* and *Ch*. *zofingiensis* estimated at 25–35 mg g^−1^ and 1–2 mg g^−1^, respectively (Kim et al. 2006; Chen et al. 2009). So, a high amount of astaxanthin produced for aquaculture could result in high consumption of microalgae biomass, electricity, water, chemicals, and labor work. Contamination of algal biomass is another important factor in challenges facing microalgae production. Microalgae cultivation may accumulate many heavy metals in biomass, further threatening the safety of the cultured fish diet. This pollution originated from using of wastewater in the cultivation of microalgae to decrease the cost of producing astaxanthin (Kang et al. 2006; Suresh Kumar et al. 2015; Ledda et al. 2016). As well, industrialization is the cause of heavy metal pollution in rivers, lakes, and underground water, highlighting the hazards of heavy metal pollution in microalgae growth. Owing to this pollution, the physical characteristics of the microalgae cells are negatively affected (Suresh Kumar et al. 2015; Lu et al. 2020).

Furthermore, oxidation occurring during storage of feed containing astaxanthin has deleterious issues, owing to the high sensitivity of astaxanthin to oxidation from the presence of a highly unsaturated molecular structure. Hence, astaxanthin depletion will further boost during long-term storage (Takeungwongtrakul and Benjakul 2016; Kasprzak et al. 2020). Besides, deleterious compounds (e.g., heptanal, heptane, nonanal, hexanal, and 2-butenal) are produced from the oxidation of lipid-soluble astaxanthin in animal feed, resulting in reducing of lipid quality during storage, which will negatively cause the nutrients loss of fish feeds (Kasprzak et al. 2020; Lu et al. 2021).

### Functional benefits of astaxanthin in aquaculture

**Fig. 5:**
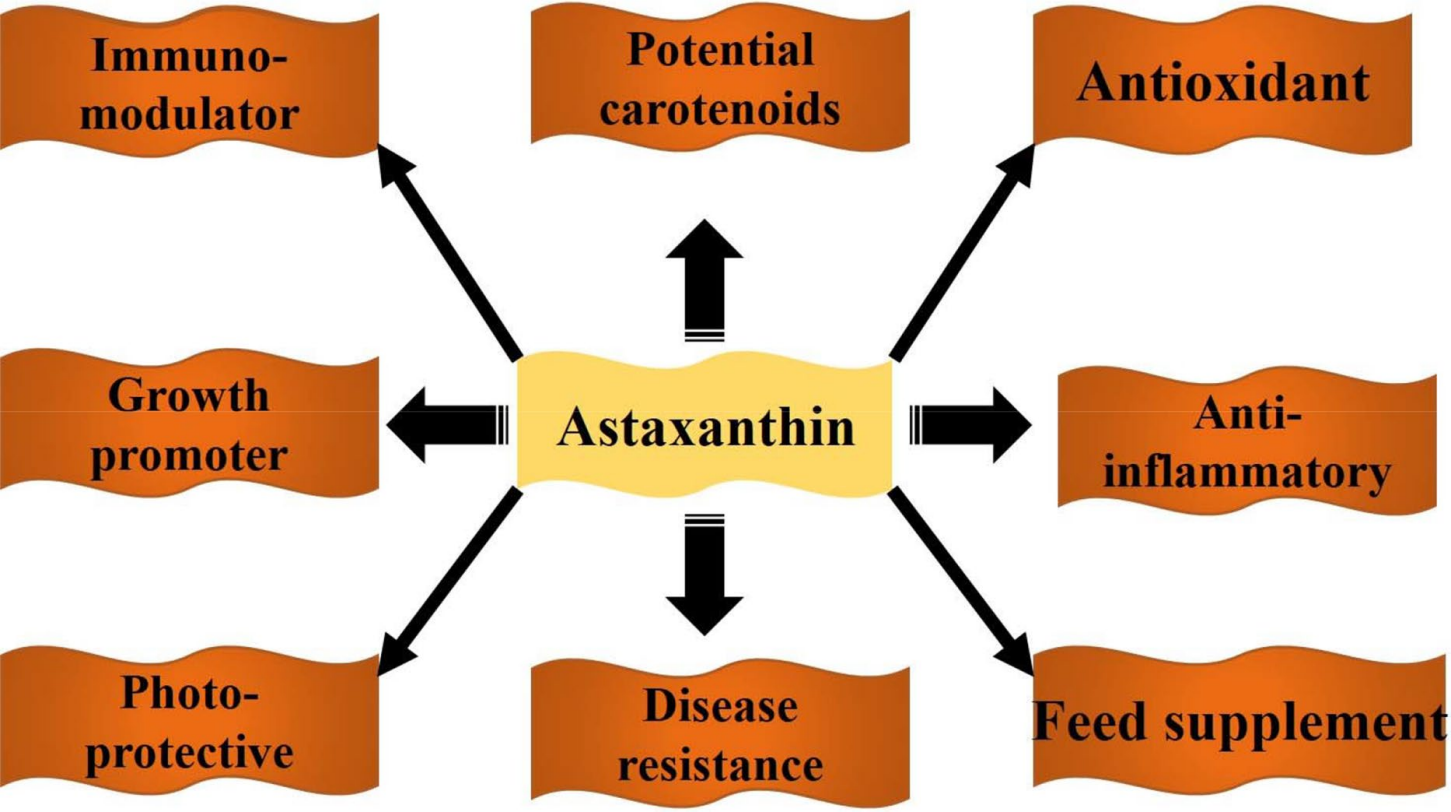
Potential health benefits of astaxanthin on aquatic animals

The biological activities of astaxanthin and its health benefits on cultured fish are displayed in Fig. 5.

## Astaxanthin is a potential carotenoid in different aquatic animals

It is recognized that the coloration of fish is a crucial quality criterion required by consumers to evaluate the health status, nutritive value, degree of freshness, and taste of farmed fish and crustaceans. In parallel, the marketing value of marine fish (e.g., salmon, red porgy, or red sea bream) depends on the color of the fish as well as the exoskeleton and muscular epithelium of shrimps, lobster, and other crustacean carapaces and molluscan gonads (EFSA 2014; Lim et al. 2018). Therefore, the external characteristics should be managed, giving paramount value to cultured aquatic animals (De Carvalho and Caramujo 2017). Besides, the quality of marine fish markedly depends on skin and muscle pigmentation in the market, thus providing humans with high-quality fish meat products. Astaxanthin is a widely distributed and highly valued carotenoid that is responsible for the pink-red color in the fins, skin, muscle, and gonads in aquatic animals, including zooplankton, marine fish, and crustaceans (Maoka 2011). Most aquatic animals (e.g., salmon, red sea bream, rainbow trout, ornamental fish, crayfish, lobster, and shrimp) cannot biosynthesize astaxanthin de novo; thus, the entrance point of astaxanthin is through dietary ingestion of higher heterotrophic zooplanktons or acquired in formulated artificial diet (Rajasingh et al. 2006). Curiously, astaxanthin retention in fish is significantly promoted in the presence of dietary lipids; this may lead to higher astaxanthin concentrations in fish muscle. This results from the fact that astaxanthin is esterified with fatty acids to intensify the storage of lipids; meanwhile, free astaxanthin may be incorporated into cell membranes, preventing lipid peroxidation and protecting membrane structures (Sommer et al. 2006; McNulty et al. 2007). In addition, dietary astaxanthin and lipids may enhance the digestion, absorption, and transport mechanisms of the digestive systems of fish (Castenmiller and West 1998). When rainbow trout were fed natural astaxanthin from *H*. *pluvialis* algae and synthetic astaxanthin (75 mg kg^−1^ of feed) using fish oil or olive oil for 6 weeks, higher serum concentrations of astaxanthin, as well as muscle astaxanthin levels, were higher in fish supplemented with synthetic carotenoid and olive oil (Choubert et al. 2006). Previous studies have recommended that dietary natural astaxanthin from microorganisms is better than synthetic one because of its superior bio-accumulation in teleost skin and muscle (Sigurgisladottir et al. 1994; Kurnia et al. 2007; Lu et al. 2021).

Given its unique properties, the progressive expansion of natural astaxanthin application as a feed additive in the aquaculture industry has been acknowledged as an insatiable instance. Numerous previous studies determined the effect of dietary natural or/and artificial astaxanthin on the skin and muscle pigmentation of various aquatic animals that have been categorized in Table 2.

**Table 2:** The impact of dietary astaxanthin on the pigmentation of aquatic animals

Species	Inclusion dose	Source	Response	References
Rainbow trout (*Oncorhynchus mykiss*)	75 mg kg^−1^	Algal and synthetic	Increased serum astaxanthin concentration, muscle astaxanthin retention, and muscle color	Choubert et al. (2006)
	100 mg kg^‒1^	Algal and synthetic	Elevated levels of astaxanthin in flesh tissue and improved coloration	Choubert and Heinrich (1993)
	100–200 mg kg^−1^	Synthetic	Increased serum astaxanthin concentration, muscle astaxanthin retention, and muscle color	Choubert et al. (2009)
	100 mg kg^−1^	Synthetic	High levels of astaxanthin in muscle tissue, improved coloration	Choubert (2010)
	40 mg kg^−1^	Algal and synthetic	Increased coloration in trout flesh and skin	Sommer et al. (1991)
	50–100 mg kg^−1^	Synthetic	High levels of astaxanthin in muscle tissue, improved coloration	Rahman et al. (2016)
	50–200 mg kg^−1^	Synthetic	Higher concentrations of astaxanthin in skin and muscles Improved skin pigmentation	NOORI and Alireza (2018)
Gilthead seabream (*Sparus aurata*)	40 mg kg^−1^	Algal and synthetic	Increased total carotenoid content in skin No effect on skin and muscle pigmentation	Gomes et al. (2002)
Red sea bream (*Pagrus major*)	30 mg kg^−1^/12 weeks	Microbial and synthetic	Enhanced the skin pigmentation	KURNIA et al. (2007)
Olive flounder, *Paralichthys olivaceus*	100–200 mg kg^−1^	Algal and synthetic	Improved skin pigmentation	Pham et al. (2014)
Large yellow croaker, *Larimichthys croceus*	75 mg kg^−1^	Synthetic	Enhanced the reddish coloration in ventral and dorsal skins Higher carotenoid content in skin	Yi et al. (2014)
Discus fish (*Symphysodon* spp.)	50–400 mg kg^−1^	Synthetic	Improved the skin pigmentation	Song et al. (2017)
Pacific white shrimp, *Litopenaeus vannamei*	500 mg kg^−1^	Algal	Increased red-color pigmentation in exoskeleton and muscle	Parisenti et al. (2011)
	25–50 mg kg^−1^	Synthetic	Higher astaxanthin content of shrimp shell	Zhang et al. (2013)
	25–150 mg kg^−1^	Algal and synthetic	Greater pigmentation efficiency Improved tail muscle coloration	Ju et al. (2011)
	100–200 mg kg^−1^	Synthetic	Higher levels of astaxanthin content	Salarzadeh and Rajabi (2015)
Giant tiger shrimp, *Penaeus monodon*	25–100 mg kg^−1^	Synthetic	Enhanced shell coloration	Wade et al. (2015b)
Kuruma shrimp, *Marsupenaeus japonicus*	50–400 mg kg^−1^	Synthetic	Improved shell coloration	Yamada et al. (1990)
	50–100 mg kg^−1^	Synthetic	Enhanced flesh and shell pigmentation	Chien and Shiau (2005)
	400–1600 mg kg^−1^	Synthetic	Greater red pigmentation in the cephalothorax	Wang et al. (2018a)
Peppermint shrimp, *Lysmata wurdemanni*	500–1500 mg kg^−1^	Synthetic	Improved egg pigmentation	Díaz-Jiménez et al. (2019)
Atlantic salmon, *Salmo salar*	84.2 mg kg^−1^ 15 weeks	Algal	Pink coloration of muscle	Sigurgisladottir et al. (1994)
	2.1–41.4 mg kg^−1^	Synthetic	Enhanced integument and flesh pigmentation	Wathne et al. (1998)
	45 mg kg^‒1^	Synthetic	Elevated levels of astaxanthin in flesh tissue and improved coloration	Baker et al. (2002)
	12.5–50 mg kg^−1^	Synthetic	Increased astaxanthin levels in muscle and skin	Ytrestøyl and Bjerkeng (2007)
Juvenile red abalone (*Haliotis rufescens*)	300 mg kg^−1^	Synthetic	Minor shell color change	Canales-Gómez et al. (2010)
Red king crab (*Paralithodes* *camtschaticus*)	380 mg kg^−1^	Algal	Improved shell pigmentation	Daly et al. (2013)
Chinese mitten crab, *Eriocheir sinensis*	30–120 mg kg^−1^	Algal	Improved the redness of carapace and hepatopancreas	Jiang et al. (2020)
Blood parrot (*Cichlasoma citrinellum* × *Cichlasoma synspilum*)	400 mg kg^−1^	Algal	Elevated concentrations of astaxanthin in skin and scales Enhanced skin coloration	Li et al. (2018)
Red porgy (*Pagrus pagrus*)	100 mg kg^−1^	Algal	Enhanced skin coloration	Chatzifotis et al. (2005)
	25–50 mg kg^−1^	Algal and synthetic	Enhanced skin coloration	Tejera et al. (2007)
	3300 mg kg^−1^	Algal	Enhanced skin coloration	Chatzifotis et al. (2011)

## Benefits of astaxanthin on the reproduction of aquatic animals

**Fig. 6:**
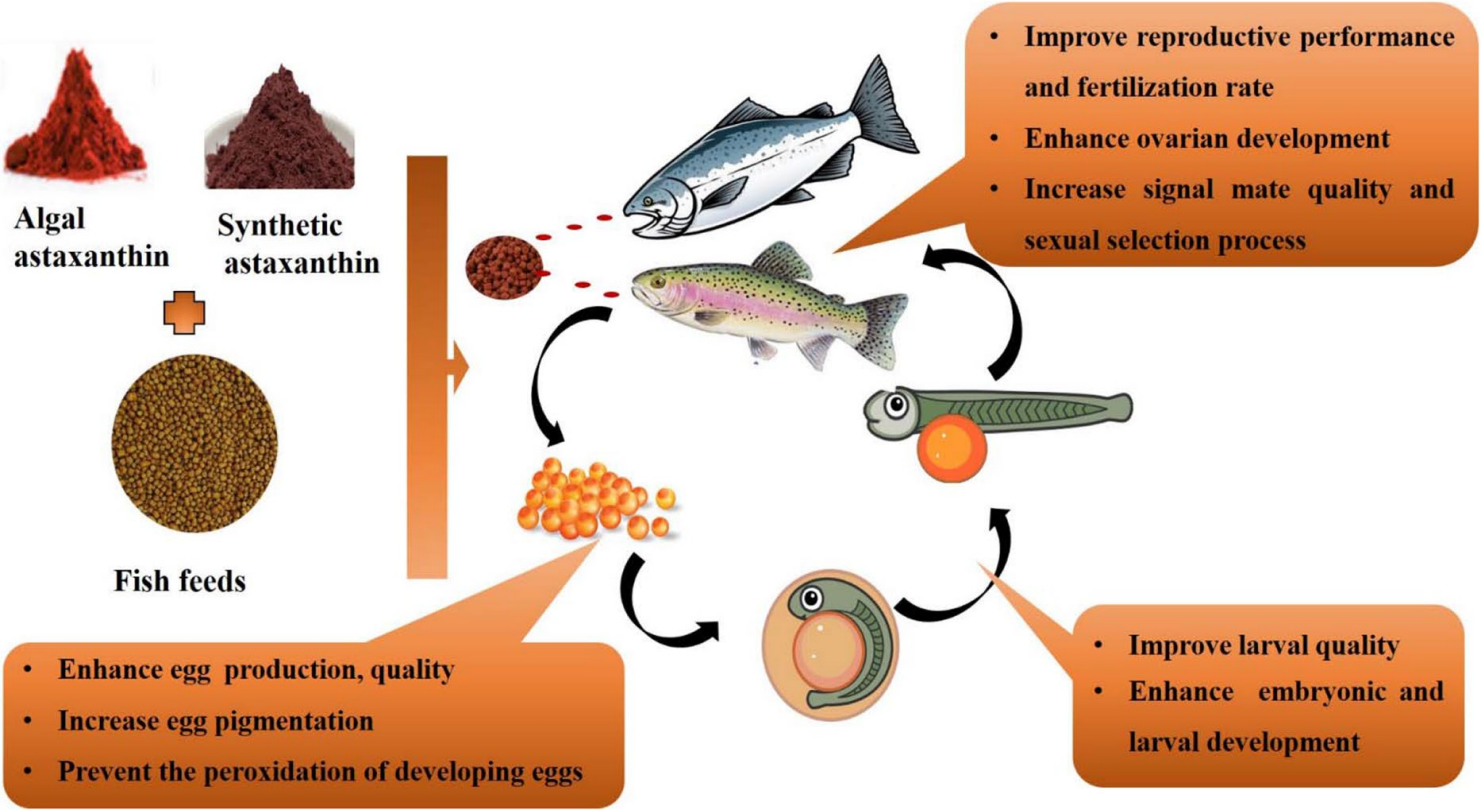
Summary of benefits of astaxanthin supplementation on different maturity stages of salmon and rainbow trout species during reproduction

A fundamental role of astaxanthin in reproductive performance, including egg production and quality, has been evidenced in many aquatic animals (Fig. 6) (Tizkar et al. 2013, 2015; Palma et al. 2017). Supplemented carotenoids consumed by aquatics are accumulated in the liver and crustaceans’ hepatopancreas which are then transferred to the ovaries in the late stages of maturity (Harrison 1990, 1997; Tizkar et al. 2013).

Besides, most of the reproductive activities of aquatic organisms are mediated by astaxanthin via its accumulation within reproductive organs, such as observed in salmonid fish during sexual maturation, pooling from the muscle and liver into the gonads that concentrated in the unesterified form in the mature eggs, which its coloration is considered an indicator about egg quality (Blount et al. 2000; Rajasingh et al. 2006; Nie et al. 2011; Wade et al. 2015a).

One of the most coordinated effects of astaxanthin is its function as a primary source of vitamin A precursors (retinol and 3,4-didehydroretinol) (Torrissen 1990; Moren et al. 2002; Blomhoff and Blomhoff 2006), which they have a substantial role in cell signaling during the shaping of developing vertebrate embryos (Duester 2008; Kam et al. 2012). For this reason, it plays a crucial role in the increment of vitamin A concentration in feeding fry of Atlantic salmon, *Salmo salar* (Christiansen et al. 1994), as well as these investigations have been observed in the intestines of rainbow trout and Atlantic salmon (White et al. 2003). The impact of dietary astaxanthin supplementation on the breeding behavior of a variety of fish species has been documented in many previous studies. Dietary inclusion of astaxanthin (0.07, 12.46, 33.33, 65.06, and 92.91 mg kg^−1^) for six months has been regarded as an indispensable supplement for effective reproductive traits as this carotenoid has enhanced the oocyte maturation in the broodstock rainbow trout, *O*. *mykiss*, and fertilization rate in different maturity stages (Ahmadi et al. 2006; Bazyar Lakeh et al. 2010). Moreover, adding astaxanthin to broodstock diets had positive effects on the egg quality of yellowtail (*Seriola quinqueradiata*) (Verakunpiriya et al. 1997), striped jack (*Pseudocaranx dentex*) (Vassallo-Agius et al. 2001), and sea urchin (*Lytechinus variegatus*) (George et al. 2001).

The role of astaxanthin in enhancing the efficacy of the propagation processes and embryonic developmental stages for goldfish, which is the most precious ornamental fish in the world, has been taken great attention (Tizkar et al. 2013, 2015). Dietary synthetic astaxanthin intake (50, 100, and 150 mg kg^−1^) for 120 days has promoted the quality features of sperm (e.g. concentration, motility, osmolality) and fertility of goldfish *Carassius auratus* (Tizkar et al. 2015). In addition, the diameter and the number of fertilized eggs in the goldfish *Carassius auratus* were greater in groups fed 150 mg kg^−1^ of astaxanthin for four months, which correlated to higher egg survival rates in the incubation period (Tizkar et al. 2013). Recent investigation on the effects of astaxanthin supplementary diets (150 mg kg^−1^ feed) on egg quality and larvae quality parameters of clownfish (*Amphiprion ocellaris*) has observed a higher hatching rate of egg and survival rate with a lower malformed rate of larvae (Hue et al. 2020). It has been also claimed that in crustaceans, carotenoid supplementation was a vital process during the reproductive cycle (Liñán-Cabello et al. 2003; Liñán-Cabello and Paniagua-Michel 2004). However, marbled crayfish (*Procambarus fallax* f. *virginalis*) fed astaxanthin did not enhance maturation (Kaldre et al. 2015). The reproductive maturation, including ovarian development, has been improved in two penaeid species (*Artemesia longinaris* and *Pleoticus muelleri*) by using astaxanthin added in artificial feeds at 300 mg kg^−1^ of diet for 45 days (Díaz et al. 2020). Likewise, dietary supplementation of formulated diets with 500 mg kg^−1^ astaxanthin has significantly improved the maturation and spawning performance of *Penaeus monodon* broodstock, as especially mentioned by several eggs in gravid females and the number of spermatozoa in male shrimp (Paibulkichakul et al. 2008). In aid to this fact, astaxanthin (200 mg kg^−1^ diet) has the most effective antioxidant as it reduced malondialdehyde (MDA) levels in the ovarian tissue of narrow-clawed crayfish, *Astacus leptodactylus* (Eschscholtz), resulting in increased ovarian egg number and size (Barim-Oz and Sahin 2016), thus preventing the peroxidative damage of reproductive tissues and developing eggs that are attributable to its potent antioxidant capacity to quench excessive amounts of destructive singlet oxygen and free radicals against several stressors, involving ultraviolet light and chemical exposure and physiological stress (Britton 2008; Palozza et al. 2009; Pham et al. 2014; Lim et al. 2018).

## Benefits of astaxanthin on the antioxidant capacity, immunity, and disease resistance

The rise of infectious illnesses in intensive farming, particularly in the early phases of production, constitutes a substantial drawback or leading threat that has a considerable influence on the global economy. Significant scientific efforts have been made throughout the years to improve the immune systems and antioxidant capacity of numerous fish and crustaceans by consuming astaxanthin (AX). We have demonstrated some previous reports on the impacts of Ax on enhancing antioxidant capacity, immunity, and disease resistance against infection in different aquatic animals (Table 3). Extensive research has demonstrated that AX has many advantages for crustaceans, in addition to the typical pigmentation properties, antioxidant activity, immune system support, resistance to disease, and resistance to environmental stressors like temperature, pH, and ammonia. But since it is generally known that crustaceans cannot produce AX on their own, several species of cultured crustaceans need dietary supplements of AX (Tejera et al. 2007; Wade et al. 2017).

**Table 3:** The impact of dietary astaxanthin on the antioxidant capacity, immunity, and disease resistance

Species	Inclusion dose and duration	Source	Response	References
Rainbow trout, *Oncorhynchus mykiss*	0, 50, 75, and 100 mg kg^‒1^ For 10 weeks	Synthetic	Reduce catalase and SOD activities with higher total antioxidant status	Choubert et al. (2009)
	100 mg kg^−1^	Synthetic	Improve immune response and resistance to IHNV infection	Ameur et al. (2012)
	80 mg kg^−1^ For 16 weeks	Algal and synthetic	Microalgal docosahexaenoic acid (DHA) has more impacts than those of synthetic astaxanthin (AX) that reduced MDA and increased glutathione concentrations, in addition to upregulation of mRNA levels and activities of major redox enzymes (GR), GPX, GST, and SOD) in the muscle and liver of trout fed on a diet containing DHA or AX	Sommer et al. (1991)
*Common carp*, *Cyprinus carpio*	0, 25, 50, and 100 mg kg^−1^	Synthetic	The cumulative mortality *C. carpio* against *A. hydrophila* was reduced especially with 50 and 100 mg kg^−1^ enriched diets. The phagocytic ratio and phagocytic index as well as respiratory burst, anti-protease, lysozyme, and bactericidal activities registered a significant increase with all enriched diets, suggesting that AX can modulate the immune system	Ju et al. (2011)
Blood parrot, *Cichlasoma citrinellum/Cichlasoma synspilum*	400 mg kg^−1^ For 50 days	Algal	Pigmented fish had lower SOD, CAT, and MDA and higher TAC	KURNIA et al. (2007)
*European sea bass*, *Dicentrarchus labrax*	60, 80, and 100 mg kg^−1^ For 60 days	Synthetic	Enhance hepatic SOD and GPx as well as increase intestinal mucosal phagocytic and lysozyme activities	KURNIA et al. (2007)
Asian seabass, *Lates calcarifer*	1% inclusion level	Algal	The survival % of larvae against *V. alginolyticus* was highest, as well as SOD and catalase activities were significantly increased in AX supplemented group relative to the control	Pham et al. (2014)
Juvenile largemouth bass *Micropterus salmoides*	75, 150 mg kg^−1^ For 8 weeks	Synthetic	AX supplementation reduced MDA content and increased superoxide dismutase activity. It also reduced the mRNA levels of caspase 3, caspase 9, BAD, and IL15 in HFD-stressed fish	Xie et al. (2020)
Large yellow croaker, *Larimichthys croceus*	0.22, 0.45, and 0.89 mg kg^−1^ For 66 days	Algal	Improve serum antioxidant capacity (increased SOD, GPx, catalase activities and decrease MDA contents) and immune response (lysozyme activities and complement contents)	Yi et al. (2014)
Yellow catfish, *Pelteobagrus fulvidraco*	80 mg kg^−1^ For 60 days	Synthetic	AX increased serum lysozyme and alkaline phosphatase activities, as well as hepatic catalase, SOD activities, and hepatic HSP70 mRNA levels at 24 h after the initiation of crowding stress	Yi et al. (2014)
Olive flounder, *Paralichthys olivaceus*	100. 200 mg kg^−1^ For 8 weeks	Algal and synthetic	Liver and plasma SOD activities and radical scavenging activities in the muscle, liver, and plasma were reduced in juvenile fish	Pham et al. (2014)
Chinese mitten crab, *Eriocheir sinensis*	30, 60, 90, and 120 mg kg^−1^ For 40 days	Algal	Positive increase of SOD, GPx, TAC, lysozyme, and acid phosphatase (ACP) activities with highest mRNA levels of EsLecA, EsTrx, and EsPrx6 in hepatopancreas and hemolymph, as well as EsMyd88 and EsHc mRNA expression levels, reached the peaks in the juvenile fish	Jiang et al. (2020)
Northern snakehead*, Channa argus*	50, 100, and 200 mg kg^−1^ For 56 days	Algal	Oxidative damage induced by the LPS challenge was significantly corrected by AX supplementation via promoting the levels of antioxidant enzymes (increase GST, GSH-Rt, GPx, SOD, catalase activities and decrease MDA contents), immune parameters (elevate lysozyme, complement 3, complement 4, while reduce IL-1β and TNF-α levels) and increase expression of glucocorticoid receptor and HSP70 mRNA levels in the liver, spleen, kidney, and intestine	Song et al. (2017)
Oscar*, Astronotus ocellatus*	200 mg kg^−1^	Synthetic	Improved immunological parameters, resistance to *Aeromonas hydrophila*	Alishahi et al. (2015)
Pacific white shrimp, *Litopenaeus vannamei*	25, 50, 75, 100, 125, and 150 mg kg^−1^ For 56 days	Synthetic	AX alleviated oxidative stress by restoring cMnSOD, catalase, Hsp70, and HIF-1α mRNA expression levels toward a normal level	Zhang et al. (2013)
	1.7, 3.3, 6.7, and 13.3 g kg^−1^ For 25 days	Algal	AX diminished MDA and mRNA expression of SOD and GPx in white shrimp larval livers after salinity stress, whereas T-AOC increased. In addition, AX regulated the NF-kB pathway mRNA expression, which indicated that astaxanthin might improve the anti-inflammation and immune properties	Xie et al. (2018)
	120–150 mg kg^−1^ For 8 weeks	Synthetic	AX stimulated higher total antioxidant status and tolerance of shrimp which suffered from low dissolved oxygen stress (0.8 mg L^−1^) besides up-regulating hypoxia-inducible factor-1α (HIF-1α), cytosolic manganese superoxide dismutase (cMnSOD), and catalase mRNA expression levels	Ju et al. (2011)
	50 ppm For 56 days	Synthetic	Significant increase of hemolymph T-SOD and hepatopancreatic mRNA expression level of GPx, catalase with a lower level of hemolymph T-AOC and MDA, hepatopancreatic mRNA of anti-inflammatory ability (Relish, Rho, and HSP70) and apoptosis-related gene expression (caspase 3)	Fang et al. (2021a)
	25, 50, 100, 200 mg kg^−1^ For 8 weeks	Synthetic	Enhance the activities of SOD, catalase, GPx, and increased levels of T-AOC and GSH, in addition to decreased MDA. Upregulate the expression levels of cMn-SOD, CAT, and GPx genes and stimulate the immunity indices (hematocyte count, total protein, lysozyme, phagocytic activity, and phenoloxidase). Increase the relative percentage of survival following *Vibrio harveyi* infection	Eldessouki et al. (2022)
	80 mg kg^−1^ For 4 weeks	Synthetic	High-level resistance to white spot syndrome virus (WSSV), is associated with remarkable improvement of hemolymph immunological index, including phagocytic activity, total hemocyte count as well as phenoloxidase, anti-superoxide radical activity, bacteriolytic activity antibacterial activities. AX also promoted mRNA expression of antioxidant enzyme genes (CAT, cMnSOD, and GPx) in the hepatopancreas	Salarzadeh and Rajabi (2015)
Kuruma shrimp, *Marsupenaeus japonicus*	400, 800, 1200 mg kg^−1^ For 56 days	Synthetic	AX improved total hemocyte count, viable cells count, and the rate of phagocyte activity of juvenile shrimp	Yamada et al. (1990)
Asian tiger shrimp, *Penaeus monodon*	71.5 mg kg^−1^ For 8 weeks	Synthetic	Juveniles fed the diet supplemented with AX displayed astounding antioxidant status in stressed shrimp	Díaz-Jiménez et al. (2019)
	80 mg kg^‒1^ For 8 weeks		AX exhibited enhanced antioxidant defense capability (SOD) and better hepatopancreatic function (lower hemolymph ALT and AST)	Chien et al. (2003)
	200–300 mg kg^−1^	Algal	Greater resistance to white spot syndrome virus (WSSV), while phenoloxidase activity and total hemocyte count were negatively correlated	Supamattaya et al. (2005)
Giant freshwater prawn, *Macrobrachium rosenbergii*	0.67 and 1.34 nmol g^−1^ via injection	Synthetic	Improved survival and resistance against *Lactococcus garvieae* infection	Angeles Jr et al. (2009)

The availability and synchronization of intrinsic and extrinsic antioxidants are necessary for fishes’ antioxidant defenses to scavenge free radicals such as reactive oxygen species (ROS) (Ju et al. 2011). Catalase, superoxide dismutase (SOD), glutathione (GSH), glutathione reductase (GR), glutathione S-transferase (GST), and glutathione peroxidase (GPX) are examples of intrinsic or endogenous antioxidant defenses (Sommer et al. 1991). With reducing and nucleophilic characteristics, GSH is the main cellular thiol that is not a protein (Pisoschi and Pop 2015). The antioxidant enzyme GPx, which may destroy the lipid peroxide (LPO, MDA) produced within cells, is known to use GSH as a substrate (Livingstone 2001). Both environmental stressors and infection can increase ROS and MDA production and reduce antioxidant molecules such as GSH, SOD, and catalase (Pan et al. 2011; Ameur et al. 2012; Ho et al. 2013; Srikanth et al. 2013; Regoli and Giuliani 2014; Dawood et al. 2020b).

The outstanding antioxidant properties of synthetic and algal AX are a strong pro-oxidant due to its exceptionally high unsaturation and oxidative potential in the chemical structure (Ismail et al. 2016). Indeed, AX has 100 times more antioxidative activity than tocopherol and beta-carotene, respectively (Mularczyk et al. 2020). AX possesses keto (= O) and hydroxyl (–OH) groups, conjugated carbon–carbon double bonds, and both lipophilic (hydrophobic, non-polar) and hydrophilic (polar) characteristics. Polar functional groups of AX are orientated outside the membrane, and the hydroxyl groups are attached across membranes (Kishimoto et al. 2016; Britton 2020). The AX backbones could act as molecular wire to strengthen the membrane's mechanical properties (Pashkow et al. 2008; Skibsted 2012). When compared to control fish fed a non-AX diet, fish fed AX showed a marked reduction in MDA levels in many tissues (Kurnia et al. 2007). MDA is a good marker of tissue oxidative stress-related injury in the body (Kaneda and Miyazawa 1987). Additionally, fish and invertebrates have been shown to produce more antioxidative enzymes like SOD, catalase, and GPx as well as cellular endogenous antioxidants like GSH when carotenoids like AX are administered (Pan et al. 2011; Sahin et al. 2014; Al-Amin et al. 2015; Gammone et al. 2015; Lim et al. 2018; Dawood et al. 2020a). As well, AX supplementation could increase serum TAC levels in different aquatic animals (Kurnia et al. 2007).

Additionally, it has been discovered that the addition of carotenoids increases the expression of HSP in cultured cells and the tissue of several animal species (Müller et al. 2013; Cheng et al. 2018; Fleischmann et al. 2020; Tan et al. 2020). Carotenoids demonstrated antioxidant action may work not only by directly scavenging ROS but also by regulating the expression of proteins involved in stress and antioxidant defenses. The three following reactions, electron transfer (oxidation and reduction), hydrogen abstraction (allylic hydrogen atom abstraction), and radical addition (adduct formation), are assumed to be the three processes through which carotenoids interact with free radicals (Pashkow et al. 2008; Sahin et al. 2014; Gammone et al. 2015; Nishino et al. 2016). Carotenoids’ mechanisms of action in the body have been divided into the following four groups: Antioxidative and pro-oxidative actions, reduction of NF-kB signaling translation, activation of the nuclear factor erythroid 2-related factor 2 (Nrf2), and interaction with other transcription factors are all examples of these effects (Kaulmann and Bohn 2014; Niu et al. 2018; Rodriguez-Concepcion et al. 2018; Dawood et al. 2020a). It is well recognized that transcription factors like NF-kB and Nrf2 are connected to immunological response, inflammation, and oxidative stress responses. Inflammatory substances like tumor necrosis factor (TNF) and cytokines, as well as oxidative stress, activate the NF-kB pathway. On the other hand, it is known that the Nrf2 pathway plays a significant role in cells' defense against ROS-induced oxidative stress (Kaulmann and Bohn 2014). The inducible genes Nrf2, heme oxygenase 1 (HO-1), and iNOS, which control inflammatory responses and oxidative stress, are transcribed by the protein NF-kB, which is inhibited by AX in aquatic and different animals-(Kaulmann and Bohn 2014; Xie et al. 2018; Le Goff et al. 2019; Jiang et al. 2020). Moreover, AX supplementation in aquaculture can suppress inflammatory cytokines and apoptotic markers such as caspase 3, caspase 9, and IL-15, IL-1*β* and TNF-*α* levels (Song et al. 2017). Together, these findings imply that dietary carotenoids can enhance the body’s natural antioxidant defenses, such as antioxidant enzymes, cellular endogenous antioxidants, and HSP, as well as increase resistance to oxidative stress and suppress inflammatory and apoptotic cascade.

Enhanced phagocytic activity in fish has been documented after treatment with diverse AX against different infections (Harikrishnan and Balasundaram 2005; Sahu et al. 2007; Harikrishnan et al. 2010b, 2011). Since O_2_^−^ is the first product released during the respiratory burst, O_2_^−^ concentration has been accepted as an accurate parameter to quantify the intensity of a respiratory burst (Secombes 1990). The overall proportion of NBT-positive blood cells remained stable between increasing in rainbow trout following immunostimulant therapy (Jeney and Anderson 1993). These cells could be neutrophils that are still capable of the creation of reactive oxygen species. With all doses of the pathogen-specific supplemental diets, the serum lysozyme activity was significantly increased. Fish treated with herbal remedies containing AX for various illnesses have shown increased serum lysozyme activity (Martins et al. 2002; Harikrishnan and Balasundaram 2008; Harikrishnan et al. 2009, 2010a). Increasing trends in serum lysozyme activity may have contributed to the improvement in non-specific defensive mechanisms described in several fish against pathogens (Sahu et al. 2007). Common carp, *Cyprinus carpio*, treated with 50 and 100 mg kg^−1^ of AX supplemental diet against pathogens showed a considerable increase in serum phagocytic, respiratory burst, lysozyme, and bactericidal activities that led to a decrease in the proportion of death during the first 30 days after *Aeromonas hydrophila* infection (Ju et al. 2011). Many previous reports reported that AX directly or indirectly confers antioxidant activity and enhances innate, cell-mediated, and humoral immune responses (Jyonouchi et al. 2000; Kurihara et al. 2002; Park et al. 2011). The impacts of AX on innate immunity may be correlated to its ability to trigger further antimicrobial effects processes, such as lysosomal enzyme release, complementary elements, cationic peptides, and the synthesis of oxygen reactive species (Chew et al. 2011; Smith et al. 2013).

## Benefits of astaxanthin on growth and stress tolerance

Astaxanthin has great attention nowadays due to its numerous physiological actions in aquatic animals (Lim et al. 2018; Lu et al. 2021). Carotenoids may promote great nutrient use, resulting in improved growth performance in many aquatic species (Amar et al. 2001). For instance, adding astaxanthin to the diet may improve the growth of certain species, such as *Micropterus salmoides* (Xie et al. 2020), *Trachinotus ovatus* (Fang et al. 2021b), *Marsupenaeus japonicas* (Wang et al. 2018a), and *Paralithodes camtschaticus* (Daly et al. 2013). Additionally, adding carotene to the food improved the growth abilities of *Oreochromis niloticus* (Hu et al. 2006), *Piaractus mesopotamicus* (Bacchetta et al. 2019), and *Penaeus monodon* (Niu et al. 2014). There were three basic explanations for how carotenoid pigments could enhance crustacean growth. Firstly, the carotenoid pigment may control aquatic animals’ metabolisms via increased digestive enzyme activity, which in turn promoted nutritional digestion, absorption, and utilization, leading to increased feed intake (Baron et al. 2008; Zhang et al. 2013). Secondly, the carotenoid pigment may also shorten the time between molt cycles in crustaceans and regulate the NADPH metabolism, both of which reduce energy consumption and improve growth performance (Hertrampf and Piedad-Pascual 2003; Mao et al. 2017). The last hypothesis is the ability of astaxanthin to enhance intestinal flora to break down indigestible components to extract more nutrients (Vasudevan et al. 2006). A previous report confirmed that red porgy (*Pagrus pagrus*) fed diets containing AX had significantly lower lipid percentages, which in turn improved lipid utilization and supplied extra energy to improve growth performance (Kalinowski et al. 2011).

The immoderate stress contributes to bodily physiological malfunction, growth rate decrease, immunological suppression, susceptibility to pathogenic invasions, and even mortality (Ndong et al. 2007; Nikoo et al. 2010; Liu et al. 2016). Therefore, it is crucial in aquaculture research to alleviate adverse conditions that could lead to significant stress and impair the host organism. AX has been reported to increase growth and resistance against stressors in many aquatic animals (Table 4). The ability of AX in crustaceans and fish diets to enhance stress resistance is attributed to its increased antioxidant capacity and immune response (Lim et al. 2018). However, under hypoxic conditions, shrimp-fed diets containing AX or beta-carotene had higher levels of HIF-1 mRNA expression, suggesting that dietary AX or beta-carotene may help partially reduce the hypoxia stress response by improving the effectiveness or utility of the oxygen transportation (Niu et al. 2014). It would have been possible for excessively high numbers of oxygen radicals to form under thermal, salinity, and osmotic stresses (Lim et al. 2018). AX may scavenge oxygen radicals in cells and lessen cellular damage and boost resistance because it has a lengthy conjugated double-bond structure and relatively unstable electron orbitals (Chien and Shiau 2005).

**Table 4:** The impact of dietary astaxanthin on growth and stress tolerance

Species	Inclusion dose	Source	Response	References
Rainbow trout, *Oncorhynchus mykiss*	50,100, 200 mg kg^−^1	Synthetic	Enhanced growth and survival	Choubert et al. (2009)
	12.5, 92.9 mg kg^−1^	Synthetic	Promoted growth rate	Bazyar Lakeh et al. (2010)
Common carp fingerlings, *Cyprinus carpio*	100 and 200 ppm concentrations	Synthetic	Higher resistance to ammonia stress	Ju et al. (2011)
*European sea bass*, *Dicentrarchus labrax*	60, 80, and 100 mg kg^−1^	Synthetic	Promoted growth performance and increased resistance to osmotic stress	KURNIA et al. (2007)
Asian seabass, *Lates calcarifer*	1% inclusion level	Algal	Boost weight gain	Pham et al. (2014)
Atlantic cod, *Gadus morhua*	50, 100 mg kg^−1^	Synthetic	Enhanced growth rate	Hansen et al. (2016)
Large yellow croaker, *Larimichthys croceus*	0.22, 0.45, and 0.89 mg kg^−1^	Algal	Boosted weight gain and improved growth	Yi et al. (2014)
Yellow catfish, *Pelteobagrus fulvidraco*	80 mg kg^−1^	Synthetic	Improved resistance to acute crowding stress	Yi et al. (2014)
Red king crab, *Paralithodes* *camtschaticus*	380 mg kg^−1^	Algal	Stimulated growth performance	Daly et al. (2013)
Juvenile Chinese mitten crab, *Eriocheir sinensis*	30, 60, 90, and 120 mg kg^−1^	Algal	Lower mortality rates against ammonia-N stress	Jiang et al. (2020)
Oriental river prawn, *Macrobrachium nipponense*	50–150 mg kg^−1^	Synthetic	Improved resistance to chemical and physical stress	Tizkar et al. (2014)
Characin, *Hyphessobrycon eques*	5–20 mg kg^−1^	Synthetic	Improved resistance to ammonia stress, enhanced antioxidant status	Pan et al. (2011)
Peppermint shrimp *Lysmata wurdemanni*	400 mg kg^−1^	Synthetic	Boosted weight gain and improved growth rate	Kurnia et al. (2007)
Pacific white shrimp, *Litopenaeus vannamei*	25, 50, 75, 100, 125, and 150 mg kg^−1^	Synthetic	AX supplementation enhanced low DO stress tolerance (Increase survival rate %, especially with higher doses, 125 and 150 mg kg^−1^)	Zhang et al. (2013)
	100, 400 g kg^−1^	Algal	Higher tolerance to low dissolved oxygen	Niu et al. (2009)
	120–150 mg kg^−1^	Synthetic	More tolerance of shrimp that suffered from low dissolved oxygen stress (0.8 mg L^−1^)	Ju et al. (2011)
	1.7, 3.3, 6.7, and 13.3 g kg^−1^	Algal	improved endurance to salinity stress	Xie et al. (2018)
	80 mg kg^−1^	Synthetic	Higher tolerance to low salinity stress conditions and improved hematological responses	Flores et al. (2007)
	25, 50. 100, 200 mg kg^−1^	Synthetic	Stimulated the final weight, weight gain, and specific growth rate	Eldessouki et al. (2022)
	50 ppm	Synthetic	Significantly higher weight gain rate and specific growth rate	Fang et al. (2021a)
Kuruma shrimp, *Marsupenaeus japonicus*	50 and 100 mg kg^−1^	Algal and synthetic	Higher tolerance to low dissolved oxygen	Chien and Shiau (2005)
	400, 800, 1200 mg kg^−1^	Synthetic	Enhanced growth performance, weight gain, and specific growth rate	Yamada et al. (1990)
Asian tiger shrimp*, Penaeus monodon*	71.5 mg kg^−1^	Synthetic	Higher resistance to different levels of ammonia stress (0.02, 0.2, 2, 20 mg L^−1^)	Díaz-Jiménez et al. (2019)
	80 mg kg^‒1^	Synthetic	Subsequent improvement recovery against osmotic and thermal stresses	Chien et al. (2003)
	200–300 mg kg^−1^	Algal	More tolerable to hypoxic conditions (0.8–1 mg L^−1^)	Supamattaya et al. (2005)
	100 mg kg^−1^	Synthetic	Boosted weight gain and improved survival against hypoxia condition	Niu et al. (2014)

## Pharmacokinetic properties of astaxanthin on human health concerning its bioavailability

Like most marine carotenoids, astaxanthin is generally absorbed by the organisms along with fatty acids via passive diffusion into the intestinal epithelium, where astaxanthin mixes with bile acid and assembles into micelles. Then, these micelles are partly absorbed by intestinal mucosal cells, which are combined with chylomicrons to be delivered to the liver. After that, astaxanthin is assimilated with lipoproteins and transported to a variety of tissues (OKADA et al. 2009).

Furthermore, astaxanthin can maintain the functional integrity of cell membranes *by* placing itself in the lipid bilayers, which can protect cells, lipids, and membrane lipoproteins against oxidative damage. Once degraded, carotenoids are stored in the liver and re-secreted, either as very low-density lipoproteins (VLDL), low-density lipoproteins (LDL), or high-density lipoproteins (HDL), eventually being transported to the tissues via circulation (OKADA et al. 2009; Zuluaga et al. 2018).

The bioavailability of carotenoids relies on their chemical structures as polar-free astaxanthin has higher bioavailability than apolar β-carotene and lycopene (Bohn 2008; Yuan et al. 2011). Astaxanthin from *H*. *pluvialis* has also shown better bioavailability than *β*-carotene from *Spirulina platensis* and lutein from *Botryococcus braunii* (Ranga Rao et al. 2010). Furthermore, *cis*-astaxanthins have more preferential accumulation in the blood plasma compared with the trans-form in respect of evident shorter chain lengths (Bohn 2008; Yuan et al. 2011). However, there was a scientific controversy study that declared that xanthophyll esters such as astaxanthin were hydrolyzed in the small intestine for absorptive mechanism in humans as the enzymatic esterification of astaxanthin in the intestinal cells has a lower activity (Sugawara et al. 2009). Subsequently, the esterified astaxanthins were incorporated into the lipid core in chylomicron and loaded into a variety of human tissues. It was important to clarify that astaxanthin esters from *H*. *pluvialis* increased the bioavailability of astaxanthin as polar xanthophylls were preferable for esterification in intestinal cells to get more acknowledge about the absorption, metabolism, and biological function of polar carotenoids (Sugawara et al. 2009; Ranga Rao et al. 2010).

Many clinical studies have reported that even higher doses of natural or synthetic astaxanthin during the treatment trial period revealed harmless and non-toxic effects (Kupcinskas et al. 2008; Yuan et al. 2011; Donoso et al. 2021). Previous studies documented astaxanthin bioavailability in human plasma after administration of natural and synthetic sources of astaxanthin (Østerlie et al. 2000; OKADA et al. 2009). Astaxanthin absorption in humans was also promoted by lipid-based formulations as carotenes can solubilize into the oil phase of the food matrix with greater bioavailability and absorption capability (Olson 2004).

Given its unique health benefits, astaxanthin has widely associated with neuroprotective, cardioprotective, and antitumoral properties, proposing its ameliorative potential for the prevention of diseases like dementia, Alzheimer, Parkinson, and cardiovascular diseases. Besides, promising results would be applied to skin and eye health, highlighting its perspective on the prevention of skin *photo-aging* and the eye diseases like glaucoma, cataracts, and uveitis (Dhankhar et al. 2012; Ambati et al. 2014; Donoso et al. 2021). Astaxanthin exerts beneficial effects on pancreatic B-cell function related to antidiabetic activity via diminishing hyperglycemia in these cells and improving glucose tolerance and serum insulin levels (Uchiyama et al. 2002). Some data on human trials regarding the role of astaxanthin in the immune response were also conducted (Andersen et al. 2007; Macpherson et al. 2008; Park et al. 2010).

## Conclusion

The roles of astaxanthin are emerging into the limelight owing to its great advantages in the aspects of the aquaculture industry for fish and crustacean rearing. All available data, i.e., use of astaxanthin as a feed supplement in cultured aquatic species, supports the conclusion that astaxanthin reveals no contraindication for aquatic animals’ nutrition. The reported data have been devoted to the screening of astaxanthin in pigmentation and the weight gain, immunity enhancement, inflammatory response, and disease resistance of economic fish and crustaceans. Besides, the previous findings discussed in this review suggest that astaxanthin may be a promising candidate for the prevention of several diseases associated with oxidative stress in aquatic animals. It is expected that with astaxanthin, aforementioned benefits of astaxanthin will be a promising and safe bio-product for the sustainable development of aquaculture in the prospective future. However, the production of astaxanthin for aquaculture feeds estimated in the range of tons must face several challenges that include the application of highly rated standards in quality and safety issues and production costs.

## Data Availability

Not applicable.

## Abbreviations

AX: Astaxanthin
H: Pluvialis Haematococcus pluvialis
ORAC: Oxygen radical absorbance capacity
GM: Genetic modification
pds: Phytoene desaturase
MDA: Malondialdehyde
GSH: Reduced glutathione
SOD: Superoxide dismutase
CAT: Catalase
GR: Glutathione reductase
GST: Glutathione S-transferase
GPX: Glutathione peroxidase
LPO: Lipid peroxide
ROS: Reactive oxygen species
TAC: Total antioxidant capacity
NF-kB: Nuclear factor kappa B
Nrf2: Nuclear factor erythroid 2-related factor 2
HO-1: Heme oxygenase 1
TNF-*α*: Tumor necrosis factor alpha
IL-1*β*: Interleukin-1*β*
IL-15: Interleukin-15
HSP 70: Heat shock protein 70
VLDL: Very low-density lipoproteins
LDL: Low-density lipoproteins
HDL: High-density lipoproteins
DHA: Docosahexaenoic acid
IHNV: Infectious hematopoietic necrosis virus
ACP: Acid phosphatase
EsLecA: *Eriocheir sinensis* Lactin A
EsTrx: *Eriocheir sinensis* Thioredoxin
EsPrx6: *Eriocheir sinensis* Peroxiredoxin 6
HIF-1α: Hypoxia-inducible factor-1α
cMnSOD: Cytosolic manganese superoxide dismutase
WSSV: White spot syndrome virus
ALT: Alanine transaminase
AST: Aspartate transaminase
